# 4,4′-Difluoro-2,2′-{[(3a*RS*,7a*RS*)-2,3,3a,4,5,6,7,7a-octa­hydro-1*H*-1,3-benzimidazole-1,3-di­yl]bis­(methyl­ene)]}diphenol

**DOI:** 10.1107/S1600536811019763

**Published:** 2011-05-28

**Authors:** Augusto Rivera, Diego Quiroga, Jaime Ríos-Motta, Michal Dušek, Karla Fejfarová

**Affiliations:** a Departamento de Química, Universidad Nacional de Colombia, Ciudad Universitaria, Bogotá, Colombia; b Institute of Physics ASCR, v.v.i., Na Slovance 2, 182 21 Praha 8, Czech Republic

## Abstract

In the crystal structure of the title compound, C_21_H_24_F_2_N_2_O_2_, the two N atoms of the imidazolidine moiety are linked to the hy­droxy groups by intra­molecular O—H⋯N hydrogen-bonding inter­actions. The crystal studied was a racemic mixture of *RR* and *SS* enatiomers. The cyclo­hexane ring adopts a chair conformation and the imidazolidine group to which it is fused has a twisted envelope conformation.

## Related literature

For related structures, see: Rivera *et al.* (2010*a* ▶,*b* ▶, 2011 ▶). For uses of di-Mannich bases, see: Mitra *et al.* (2006 ▶); Elias *et al.* (1997 ▶). For related quantum-chemical literature, see: Zierkiewicz & Michalska (2003 ▶); Zierkiewicz *et al.* (2004 ▶).
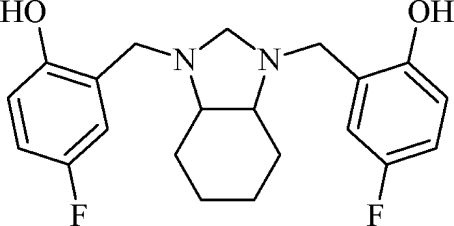


## Experimental

### 

#### Crystal data

C_21_H_24_F_2_N_2_O_2_
*M* *_r_* = 374.4
Triclinic, 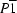

*a* = 5.4605 (1) Å
*b* = 12.4661 (3) Å
*c* = 14.3363 (4) Å
α = 108.053 (3)°
β = 91.319 (2)°
γ = 97.437 (2)°
*V* = 917.98 (4) Å^3^
*Z* = 2
Cu *K*α radiation
μ = 0.84 mm^−1^
*T* = 150 K
0.36 × 0.23 × 0.09 mm

#### Data collection

Oxford Diffraction Xcalibur diffractometer with an Atlas (Gemini ultra Cu) detector
Absorption correction: multi-scan (*CrysAlis PRO*; Oxford Diffraction, 2009 ▶) *T*_min_ = 0.516, *T*_max_ = 1
15846 measured reflections
3248 independent reflections
2819 reflections with *I* > 3σ(*I*)
*R*_int_ = 0.024

#### Refinement

*R*[*F* ^2^ > 2σ(*F* ^2^)] = 0.036
*wR*(*F* ^2^) = 0.110
*S* = 1.95
3248 reflections
250 parameters
H atoms treated by a mixture of independent and constrained refinement
Δρ_max_ = 0.25 e Å^−3^
Δρ_min_ = −0.23 e Å^−3^

### 

Data collection: *CrysAlis PRO* (Oxford Diffraction, 2009 ▶); cell refinement: *CrysAlis PRO*; data reduction: *CrysAlis PRO*; program(s) used to solve structure: *SIR2002* (Burla *et al.*, 2003 ▶); program(s) used to refine structure: *JANA2006* (Petříček *et al.*, 2006 ▶); molecular graphics: *DIAMOND* (Brandenburg & Putz, 2005 ▶); software used to prepare material for publication: *JANA2006*.

## Supplementary Material

Crystal structure: contains datablocks global, I. DOI: 10.1107/S1600536811019763/kj2178sup1.cif

Structure factors: contains datablocks I. DOI: 10.1107/S1600536811019763/kj2178Isup2.hkl

Supplementary material file. DOI: 10.1107/S1600536811019763/kj2178Isup3.cml

Additional supplementary materials:  crystallographic information; 3D view; checkCIF report

## Figures and Tables

**Table 1:** Hydrogen-bond geometry (Å, °)

*D*—H⋯*A*	*D*—H	H⋯*A*	*D*⋯*A*	*D*—H⋯*A*
O1—H1*o*⋯N1	0.88 (2)	1.92 (2)	2.7105 (15)	147.6 (19)
O2—H2*o*⋯N2	0.83 (2)	1.95 (2)	2.6975 (16)	148 (2)

**Fig. 1.**
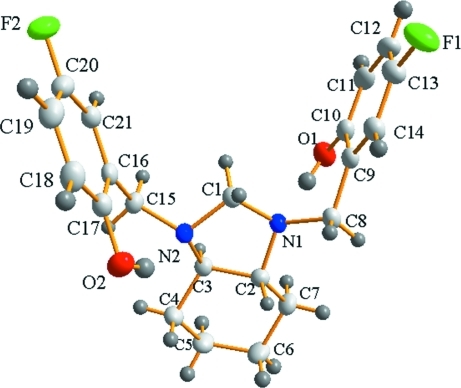
Displacement elipsoid plot of the title compound, drawn at 50% probability level.

## supplementary crystallographic information

### Comment

The title compound was obtained by a Mannich type reaction between the aminal (2*R*,7*R*,11*S*,16*S*)-1,8,10,17-tetraazapentacyclo[8.8.1.1^8,17^.0^2,7^.0^11,16^]icosane and *p*-fluorophenol. The crystal structure of the title compound was determined as a racemic mixture having (*R,R*) or (*S,S*) configurations at the two stereogenic centers and it crystallizes in a centrosymmetric space group. The chiral centers were not affected when the aminal cage reacted, so the title compound is a *trans-rac*  mixture. The molecular structure and atom-numbering scheme for the title compound are shown in Fig. 1. The crystal structure of the title confirms the presence of intramolecular hydrogen bonds between the phenolic hydroxyl groups and nitrogen atoms (Table 1). The C—O bond lengths [C10—O1, 1.3682 (17) Å; C17—O2, 1.3706 (18) Å] and the N···O distances (Table 1) are longer than the values observed in related structures where the *p*-substituents in the aromatic rings are chloride or bromide (Rivera, *et al.* 2010*b* and 2011), showing a decrease in hydrogen-bonding strength. The slight elongation of the C—O bond in the title compound could be explained by the presence of a fluorine substituent, since theoretical results using MP2 and density functional (B3LYP) methods showed that the chlorine and bromine substituents caused a shortening of this bond by a presumable contribution of these halogens in a quinoid-type structure by resonance (mesomeric) effects (Zierkiewicz,*et al.* 2003), and an electron donation from the *pz*-orbital on the oxygen atom to π* acceptor orbitals in the ring, which was not observed in *p*-fluorophenol where an inductive effect and a strong delocalization of electron density from the *pz*-orbital on the F atom to π* acceptor orbitals in the ring are predominant, leading to a suppression of electron donation from the *pz*-orbital on the oxygen atom to the aromatic ring (Zierkiewicz, *et al.* 2004).

The crystal structure showed an angular deformation in the phenol ring which is caused by the presence of the fluorine atom: the C12—C13—C14 and C19—C20—C21 internal ring angles [both 122.7 (1) °] increase by about 3.53° compared to the value of the corresponding angles in the phenol derivative (Rivera, *et al.* 2010*a*). The structural changes of the aromatic ring are governed chiefly by the electronegativity of the fluorine substituent (inductive electron withdrawal), which is reflected in an elongation of C-O bond.

### Experimental

*Physical Measurements*

The melting point was determined with an Electrothermal apparatus, and it has not been corrected. IR spectrum was recorded as KBr pellets at 292 K on a Perkin-Elmer Paragon FT—IR instrument. NMR spectra were performed in CDCl_3_ at room temperature on a Bruker AMX 400 Avance spectrometer.

*Preparation of 4,4'-Difluoro-{[2,2'-(3aRS,7aRS)-2,3,3a,4,5*,*6,7,7a-octahydro-1H-1,3- benzimidazole-1,3-diyl]bis(methylene)}diphenol*

To a solution of(2*R*,7*R*,11*S*,16*S*)-1,8,10,17-tetraazapentacyclo [8.8.1.1^8, 17^˙0^2,7^.0^11,16^]icosane (276 mg, 1.00 mmol) in dioxane (3 ml) and water (4 ml) in a two-necked round-bottomed flask, prepared beforehand following previously described procedures, was added dropwise a dioxane solution (3 ml) containing two equivalents of *p*-fluorophenol (224 mg, 2.00 mmol). The mixture was refluxed for about 6 h. The solvent was evaporated under reduced pressure until a sticky residue appeared. The product was purified by chromatography on a silica column, and subjected to gradient elution with benzene:ethyl acetate (yield 25%, m.p. = 443–447 K). Single crystals were grown from a CHCl_3_ solution by slow evaporation of the solvent at room temperature over a period of about 2 weeks.

### Refinement

All hydrogen atoms were discernible in difference Fourier maps and could be refined to reasonable geometry. According to common practice H atoms attached to C atoms were nevertheless kept in ideal positions during the refinement. The isotropic atomic displacement parameters of hydrogen atoms were evaluated as 1.2**U*~eq~ of the parent atom.

### Crystal data

**Table:**

C_21_H_24_F_2_N_2_O_2_	*Z* = 2
*M**_r_* = 374.4	*F*(000) = 396
Triclinic, *P*1	*D*_x_ = 1.354 Mg m^−^^3^
Hall symbol: -P 1	Melting point: 445 K
*a* = 5.4605 (1) Å	Cu *K*α radiation, λ = 1.5418 Å
*b* = 12.4661 (3) Å	Cell parameters from 8506 reflections
*c* = 14.3363 (4) Å	θ = 3.3–67°
α = 108.053 (3)°	µ = 0.84 mm^−^^1^
β = 91.319 (2)°	*T* = 150 K
γ = 97.437 (2)°	Prism, colourless
*V* = 917.98 (4) Å^3^	0.36 × 0.23 × 0.09 mm

### Data collection

**Table:**

Oxford Diffraction Xcalibur diffractometer with an Atlas (Gemini ultra Cu) detector	3248 independent reflections
Radiation source: Enhance Ultra (Cu) X-ray Source	2819 reflections with *I* > 3σ(*I*)
mirror	*R*_int_ = 0.024
Detector resolution: 10.3784 pixels mm^-1^	θ_max_ = 67.1°, θ_min_ = 3.3°
Rotation method data acquisition using ω scans	*h* = −6→6
Absorption correction: multi-scan (*CrysAlis PRO*; Oxford Diffraction, 2009)	*k* = −14→14
*T*_min_ = 0.516, *T*_max_ = 1	*l* = −17→17
15846 measured reflections	

### Refinement

**Table:**

Refinement on *F*^2^	90 constraints
*R*[*F*^2^ > 2σ(*F*^2^)] = 0.036	H atoms treated by a mixture of independent and constrained refinement
*wR*(*F*^2^) = 0.110	Weighting scheme based on measured s.u.'s *w* = 1/[σ^2^(*I*) + 0.0016*I*^2^]
*S* = 1.95	(Δ/σ)_max_ = 0.006
3248 reflections	Δρ_max_ = 0.25 e Å^−^^3^
250 parameters	Δρ_min_ = −0.23 e Å^−^^3^
0 restraints	

### Special details

**Table:**

Experimental. CrysAlisPro, Oxford Diffraction (2009), Empirical absorption correction using spherical harmonics, implemented in SCALE3 ABSPACK scaling algorithm.
Refinement. The refinement was carried out against all reflections. The conventional *R*-factor is always based on *F*. The goodness of fit as well as the weighted *R*-factor are based on *F* and *F*^2^ for refinement carried out on *F* and *F*^2^, respectively. The threshold expression is used only for calculating *R*-factors *etc*. and it is not relevant to the choice of reflections for refinement.The program used for refinement, Jana2006, uses the weighting scheme based on the experimental expectations, see _refine_ls_weighting_details, that does not force *S* to be one. Therefore the values of *S* are usually larger than the ones from the *SHELX* program.

### Fractional atomic coordinates and isotropic or equivalent isotropic displacement parameters (Å^2^)

**Table:**

	*x*	*y*	*z*	*U*_iso_*/*U*_eq_	
F1	0.8539 (2)	0.55112 (8)	0.62051 (7)	0.0546 (4)	
F2	−0.07887 (18)	0.52089 (7)	0.14954 (7)	0.0466 (4)	
O1	0.16405 (19)	0.16846 (9)	0.53377 (8)	0.0378 (4)	
O2	0.43069 (19)	0.15534 (9)	0.05535 (8)	0.0355 (4)	
N1	0.3641 (2)	0.10643 (9)	0.35698 (7)	0.0241 (4)	
N2	0.1999 (2)	0.09975 (9)	0.20184 (7)	0.0239 (4)	
C1	0.3018 (2)	0.17945 (11)	0.29872 (9)	0.0268 (4)	
C2	0.3651 (2)	−0.00695 (11)	0.28492 (9)	0.0240 (4)	
C3	0.1458 (2)	−0.01205 (11)	0.21678 (9)	0.0239 (4)	
C4	0.1213 (3)	−0.11590 (11)	0.12581 (10)	0.0311 (5)	
C5	0.0996 (3)	−0.22171 (12)	0.15961 (11)	0.0358 (5)	
C6	0.3115 (3)	−0.21688 (12)	0.23326 (11)	0.0367 (5)	
C7	0.3397 (3)	−0.10836 (12)	0.32279 (10)	0.0312 (5)	
C8	0.5907 (2)	0.15502 (11)	0.42136 (9)	0.0270 (4)	
C9	0.5514 (2)	0.26236 (12)	0.50104 (9)	0.0267 (4)	
C10	0.3397 (3)	0.26295 (12)	0.55485 (10)	0.0298 (5)	
C11	0.3077 (3)	0.35923 (13)	0.63185 (10)	0.0375 (5)	
C12	0.4816 (3)	0.45585 (13)	0.65482 (11)	0.0404 (5)	
C13	0.6831 (3)	0.45507 (13)	0.59942 (11)	0.0374 (5)	
C14	0.7225 (3)	0.36067 (12)	0.52322 (10)	0.0316 (5)	
C15	−0.0045 (2)	0.13892 (11)	0.15950 (9)	0.0262 (4)	
C16	0.0880 (2)	0.24381 (11)	0.13227 (9)	0.0244 (4)	
C17	0.3020 (3)	0.24648 (12)	0.08027 (9)	0.0279 (4)	
C18	0.3842 (3)	0.34067 (13)	0.05171 (10)	0.0343 (5)	
C19	0.2565 (3)	0.43379 (13)	0.07452 (11)	0.0363 (5)	
C20	0.0492 (3)	0.43006 (12)	0.12624 (10)	0.0327 (5)	
C21	−0.0371 (3)	0.33780 (11)	0.15580 (9)	0.0282 (5)	
H1a	0.449729	0.225399	0.290412	0.0321*	
H1b	0.177063	0.223466	0.329659	0.0321*	
H2	0.522627	−0.013546	0.256934	0.0288*	
H3	−0.014925	−0.022658	0.241089	0.0287*	
H4a	0.266114	−0.112776	0.089503	0.0374*	
H4b	−0.02548	−0.118546	0.086507	0.0374*	
H5a	0.095707	−0.288587	0.103492	0.0429*	
H5b	−0.055536	−0.230187	0.18856	0.0429*	
H6a	0.463729	−0.221271	0.200974	0.0441*	
H6b	0.284387	−0.282712	0.254988	0.0441*	
H7a	0.194886	−0.108455	0.359483	0.0375*	
H7b	0.486083	−0.104889	0.362647	0.0375*	
H8a	0.723214	0.172323	0.383113	0.0324*	
H8b	0.635885	0.100296	0.450802	0.0324*	
H11	0.164157	0.358531	0.669188	0.0449*	
H12	0.461937	0.522264	0.708518	0.0484*	
H14	0.86615	0.362995	0.486129	0.0379*	
H15a	−0.127904	0.156161	0.206435	0.0315*	
H15b	−0.078638	0.079531	0.101919	0.0315*	
H18	0.530423	0.341557	0.015894	0.0411*	
H19	0.311842	0.49916	0.054586	0.0435*	
H21	−0.182407	0.338481	0.192316	0.0338*	
H1o	0.188 (4)	0.1244 (17)	0.4743 (16)	0.0566*	
H2o	0.388 (4)	0.1166 (17)	0.0919 (14)	0.0533*	

### Atomic displacement parameters (Å^2^)

**Table:**

	*U*^11^	*U*^22^	*U*^33^	*U*^12^	*U*^13^	*U*^23^
F1	0.0699 (7)	0.0323 (5)	0.0487 (5)	−0.0063 (5)	−0.0012 (5)	0.0002 (4)
F2	0.0580 (6)	0.0287 (5)	0.0574 (6)	0.0137 (4)	0.0065 (4)	0.0169 (4)
O1	0.0320 (6)	0.0477 (6)	0.0311 (5)	0.0010 (5)	0.0061 (4)	0.0104 (5)
O2	0.0357 (6)	0.0400 (6)	0.0377 (6)	0.0126 (5)	0.0115 (4)	0.0187 (5)
N1	0.0253 (6)	0.0246 (6)	0.0216 (5)	0.0030 (4)	−0.0016 (4)	0.0067 (4)
N2	0.0263 (6)	0.0227 (5)	0.0227 (5)	0.0023 (4)	−0.0016 (4)	0.0079 (4)
C1	0.0307 (7)	0.0248 (6)	0.0243 (6)	0.0026 (5)	−0.0018 (5)	0.0077 (5)
C2	0.0243 (7)	0.0240 (7)	0.0236 (6)	0.0034 (5)	0.0025 (5)	0.0072 (5)
C3	0.0237 (7)	0.0244 (6)	0.0244 (6)	0.0018 (5)	0.0020 (5)	0.0095 (5)
C4	0.0357 (8)	0.0267 (7)	0.0279 (7)	0.0034 (6)	−0.0030 (6)	0.0051 (5)
C5	0.0387 (8)	0.0245 (7)	0.0404 (8)	0.0016 (6)	−0.0034 (6)	0.0065 (6)
C6	0.0394 (8)	0.0248 (7)	0.0470 (9)	0.0048 (6)	−0.0028 (7)	0.0130 (6)
C7	0.0327 (8)	0.0297 (7)	0.0344 (7)	0.0031 (6)	−0.0031 (6)	0.0155 (6)
C8	0.0249 (7)	0.0313 (7)	0.0230 (6)	0.0037 (6)	−0.0011 (5)	0.0063 (5)
C9	0.0278 (7)	0.0305 (7)	0.0212 (6)	0.0063 (5)	−0.0023 (5)	0.0068 (5)
C10	0.0287 (7)	0.0364 (8)	0.0255 (6)	0.0070 (6)	−0.0009 (5)	0.0106 (6)
C11	0.0373 (8)	0.0492 (9)	0.0266 (7)	0.0171 (7)	0.0046 (6)	0.0085 (6)
C12	0.0507 (10)	0.0369 (8)	0.0306 (7)	0.0187 (7)	−0.0013 (7)	0.0018 (6)
C13	0.0448 (9)	0.0291 (7)	0.0339 (7)	0.0023 (6)	−0.0048 (6)	0.0056 (6)
C14	0.0330 (8)	0.0332 (7)	0.0266 (7)	0.0040 (6)	−0.0007 (6)	0.0072 (6)
C15	0.0246 (7)	0.0282 (7)	0.0273 (6)	0.0030 (5)	−0.0013 (5)	0.0114 (5)
C16	0.0258 (7)	0.0272 (7)	0.0197 (6)	0.0011 (5)	−0.0041 (5)	0.0080 (5)
C17	0.0295 (7)	0.0315 (7)	0.0234 (6)	0.0042 (6)	−0.0005 (5)	0.0099 (5)
C18	0.0308 (8)	0.0423 (8)	0.0331 (7)	0.0003 (6)	0.0026 (6)	0.0189 (6)
C19	0.0409 (9)	0.0331 (8)	0.0377 (8)	−0.0027 (6)	−0.0031 (6)	0.0188 (6)
C20	0.0391 (8)	0.0259 (7)	0.0329 (7)	0.0053 (6)	−0.0039 (6)	0.0094 (6)
C21	0.0299 (7)	0.0294 (7)	0.0252 (6)	0.0040 (6)	−0.0005 (5)	0.0088 (5)

### Geometric parameters (Å, °)

**Table:**

F1—C13	1.3668 (17)	C6—H6b	0.96
F2—C20	1.3654 (18)	C7—H7a	0.96
O1—C10	1.3682 (17)	C7—H7b	0.96
O1—H1o	0.88 (2)	C8—C9	1.5100 (17)
O2—C17	1.3706 (18)	C8—H8a	0.96
O2—H2o	0.83 (2)	C8—H8b	0.96
N1—C1	1.477 (2)	C9—C10	1.4041 (19)
N1—C2	1.4704 (15)	C9—C14	1.3873 (19)
N1—C8	1.4686 (16)	C10—C11	1.3897 (18)
N2—C1	1.4805 (14)	C11—C12	1.380 (2)
N2—C3	1.4686 (18)	C11—H11	0.96
N2—C15	1.4652 (19)	C12—C13	1.371 (2)
C1—H1a	0.96	C12—H12	0.96
C1—H1b	0.96	C13—C14	1.3799 (18)
C2—C3	1.5100 (19)	C14—H14	0.96
C2—C7	1.515 (2)	C15—C16	1.507 (2)
C2—H2	0.96	C15—H15a	0.96
C3—C4	1.5151 (16)	C15—H15b	0.96
C3—H3	0.96	C16—C17	1.4032 (19)
C4—C5	1.532 (2)	C16—C21	1.388 (2)
C4—H4a	0.96	C17—C18	1.385 (2)
C4—H4b	0.96	C18—C19	1.388 (2)
C5—C6	1.531 (2)	C18—H18	0.96
C5—H5a	0.96	C19—C20	1.371 (2)
C5—H5b	0.96	C19—H19	0.96
C6—C7	1.5369 (17)	C20—C21	1.377 (2)
C6—H6a	0.96	C21—H21	0.96
			
C10—O1—H1o	106.9 (13)	H7a—C7—H7b	111.1929
C17—O2—H2o	106.8 (14)	N1—C8—C9	110.50 (11)
C1—N1—C2	105.24 (9)	N1—C8—H8a	109.471
C1—N1—C8	112.56 (10)	N1—C8—H8b	109.471
C2—N1—C8	116.09 (11)	C9—C8—H8a	109.4713
C1—N2—C3	105.27 (10)	C9—C8—H8b	109.4715
C1—N2—C15	113.08 (10)	H8a—C8—H8b	108.4269
C3—N2—C15	116.39 (10)	C8—C9—C10	119.74 (11)
N1—C1—N2	105.37 (10)	C8—C9—C14	121.22 (12)
N1—C1—H1a	109.4709	C10—C9—C14	119.04 (11)
N1—C1—H1b	109.4714	O1—C10—C9	120.76 (11)
N2—C1—H1a	109.4715	O1—C10—C11	118.91 (13)
N2—C1—H1b	109.4707	C9—C10—C11	120.31 (12)
H1a—C1—H1b	113.2798	C10—C11—C12	120.24 (14)
N1—C2—C3	100.30 (10)	C10—C11—H11	119.8818
N1—C2—C7	117.55 (11)	C12—C11—H11	119.8816
N1—C2—H2	111.2215	C11—C12—C13	118.67 (13)
C3—C2—C7	111.58 (10)	C11—C12—H12	120.6647
C3—C2—H2	117.2277	C13—C12—H12	120.6649
C7—C2—H2	99.8589	F1—C13—C12	119.05 (12)
N2—C3—C2	100.48 (9)	F1—C13—C14	118.23 (14)
N2—C3—C4	116.86 (11)	C12—C13—C14	122.72 (14)
N2—C3—H3	111.6886	C9—C14—C13	118.97 (13)
C2—C3—C4	112.03 (11)	C9—C14—H14	120.5126
C2—C3—H3	116.5452	C13—C14—H14	120.5134
C4—C3—H3	100.0584	N2—C15—C16	110.43 (10)
C3—C4—C5	107.76 (12)	N2—C15—H15a	109.4707
C3—C4—H4a	109.4714	N2—C15—H15b	109.4709
C3—C4—H4b	109.4705	C16—C15—H15a	109.4714
C5—C4—H4a	109.4712	C16—C15—H15b	109.4718
C5—C4—H4b	109.4712	H15a—C15—H15b	108.497
H4a—C4—H4b	111.1273	C15—C16—C17	119.77 (12)
C4—C5—C6	112.74 (11)	C15—C16—C21	121.39 (12)
C4—C5—H5a	109.4709	C17—C16—C21	118.82 (13)
C4—C5—H5b	109.4714	O2—C17—C16	120.69 (13)
C6—C5—H5a	109.4711	O2—C17—C18	118.90 (13)
C6—C5—H5b	109.4715	C16—C17—C18	120.40 (14)
H5a—C5—H5b	105.995	C17—C18—C19	120.43 (14)
C5—C6—C7	112.66 (13)	C17—C18—H18	119.7837
C5—C6—H6a	109.471	C19—C18—H18	119.7831
C5—C6—H6b	109.4713	C18—C19—C20	118.30 (15)
C7—C6—H6a	109.4711	C18—C19—H19	120.8492
C7—C6—H6b	109.4714	C20—C19—H19	120.8486
H6a—C6—H6b	106.0781	F2—C20—C19	119.22 (14)
C2—C7—C6	107.69 (12)	F2—C20—C21	118.08 (13)
C2—C7—H7a	109.4718	C19—C20—C21	122.70 (14)
C2—C7—H7b	109.4711	C16—C21—C20	119.33 (13)
C6—C7—H7a	109.4711	C16—C21—H21	120.3339
C6—C7—H7b	109.4711	C20—C21—H21	120.3327
			
?—?—?—?	?		

### Hydrogen-bond geometry (Å, °)

**Table:**

*D*—H···*A*	*D*—H	H···*A*	*D*···*A*	*D*—H···*A*
O1—H1o···N1	0.88 (2)	1.92 (2)	2.7105 (15)	147.6 (19)
O1—H1o···C8	0.88 (2)	2.37 (2)	2.8566 (17)	115.3 (15)
O2—H2o···N2	0.83 (2)	1.95 (2)	2.6975 (16)	148 (2)
O2—H2o···C15	0.83 (2)	2.39 (2)	2.8541 (17)	116.0 (18)
